# Phytochemicals in Chemoprevention: A Cost-Effective Complementary Approach

**DOI:** 10.7150/jca.57776

**Published:** 2021-04-30

**Authors:** Aayush Jain, Chikezie O. Madu, Yi Lu

**Affiliations:** 1 Departments of Biological Sciences, University of Memphis, Memphis, TN 38152. USA.; 2 Departments of Biological Sciences, University of Memphis, Memphis, TN 38152. USA.; 3 Department of Pathology and Laboratory Medicine, University of Tennessee Health Science Center, Memphis, TN 38163. USA.

**Keywords:** anticancer, phytochemicals, healthcare economy, targeted prevention, bioavailability

## Abstract

Cancer is one of the leading causes of death across the world. Although conventional cancer treatments such as chemotherapy and radiotherapy have effectively decreased cancer progression, they come with many dose-limiting side-effects. Phytochemicals that naturally occur in spices, fruits, vegetables, grains, legumes, and other common foods are surprisingly effective complements to conventional cancer treatments. These biologically active compounds demonstrate anticancer effects via cell signaling pathway interference in cancerous cells. In addition, phytochemicals protect non-cancerous cells from chemotherapy-induced side-effects. This paper addresses the not only the potential of phytochemicals quercetin, isoflavones, curcumin, catechins, and hesperidin in terms of cancer treatment and protection against side-effects of chemotherapy, but also methods for increasing phytochemical bioavailability.

## Introduction

### Cancer as one of the Leading Causes of Mortality Worldwide

Cancer is a major cause of mortality and is marked by the uncontrolled cell division of abnormal cells in a confined area of the body, where programmed cell death and proliferation are unbalanced. The growing health issue has caused 606,520 deaths in the U.S. in 2020 alone and is the second leading cause of death in the U.S., according to the Center for Disease Control and Prevention 1,2. In various cancer prevention and treatment stages, we review the role of phytochemicals in chemoprevention and alleviation of chemotherapy-induced side effects.

### Oncogenesis as a Complex Mechanism of Cell Transformation

Oncogenesis, where normal somatic cells are converted into cancerous cells, is a multistep process consisting of initiation, promotion, and progression, with many molecular shifts that reprogram a normal cell to experience uncontrolled cell division 3. Tumor initiation is irreversible and involves a non-fatal mutation in the cellular DNA caused by the exposure to a particular carcinogen. Following the initial exposure to a carcinogen, cellular DNA mutates aggressively 4. Initiation creates the potential for abnormal tissue growth and makes cells resistant to signals that inhibit cell division 5. If prolonged exposure continues or cells that have undergone the initiation stage proliferate, promotion occurs. After pre-cancerous cells have gotten the chance to propagate, the final stage of oncogenesis, progression, occurs where the mutations built up over time result in metastatic potential 4.

Chemoprevention refers to using specific substances during oncogenesis, biological or synthetic, to reverse or inhibit carcinogenesis before it invades pre-cancerous cells 6. During each stage of oncogenesis, phytochemicals can apply their effects in chemoprevention 7. Chemoprevention methods can be grouped into primary, secondary, and tertiary methods 8. Dietary phytochemical application is a surprisingly effective method of primary and secondary chemoprevention 8. Phytochemicals are a bioactive compound produced by plants in primary or secondary metabolism for defending plants against external dangers, thereby assisting plants' growth and reproduction. Dietary phytochemicals naturally occur in spices, fruits, vegetables, and other plant types 9. Since phytochemicals help defend plants from pathogens, they may either defend or harm non-plant organisms 10. Once a few cells are exposed to a carcinogen in the initiation stage of oncogenesis, phytochemicals can encourage the detoxification of free radicals, prompting the repair of mutated cellular DNA and eliminate initiated cells through recognizing them as foreign objects 11, 12. Even if cells have gone past the initiation stage, phytochemicals are still beneficial because they promote anti-inflammatory effects, inhibit cell proliferation, inhibit angiogenesis (development of blood vessels), and encourage apoptosis 13, 14.

### Conventional Forms of Cancer Treatment

Currently, the main cancer treatments include surgery, radiation therapy, and chemotherapy. The most prevalent drugs for chemotherapy consist of hormone targeting agents, DNA-interactive agents, and antimetabolites 15. Cancer can also be treated with therapeutic methods such as targeted therapy, immunotherapy, and hormone therapy 16. Conventionally, radiation therapy and chemotherapy are the most accepted treatment types for any cancer because of their ability to kill cancer cells at metastasizing sites, shrinking tumor sizes. However, cancer patients' responses to these remedies vary considerably for different types of cancer. An optimal cancer treatment would kill only cancer or tumor cells, but most chemotherapy forms involve a non-specific targeting of chemotherapeutic drugs to curb uncontrolled cell division and growth 17. The primary shortcomings of chemotherapy and radiotherapy are multidrug resistance, the reappearance of tumors, dose-limiting side effects in normal cells, and economic burden 18. Due to the non-specific nature of chemotherapy drugs, not all cancerous cells are always eradicated from the body, which may cause a recurrence of a tumor 19. On the other hand, phytochemicals have dual activity, meaning they exert cytotoxic effects against tumor cells while either having no effect or exerting protective effects against chemotherapy-induced side effects in normal cells. 19 Many studies have shown a lower incidence of cancer with an increase in the consumption of phytochemical-rich plant-based foods. A meta-analysis of various observational investigations established that diets with an emphasis on plant-based foods negatively correlate with cancer risk in general 20.

### Application Windows for Phytochemicals

Risk factors that play a role in cancer formation include changeable factors (e.g., environmental or dietary factors) and inborn non-changeable factors (e.g., genetic predispositions). Around 10% of all types of cancer are caused by non-changeable factors, meaning around 90% of cancer types are caused by changeable risk factors 21. Oxidizing agents and reactive oxygen species or free radicals that can be present in our food and the air lead to DNA damage. It is vital to keep a balance between the body's detoxification pathway and the intake of free radicals through our environment, illuminating the vital role of changeable risk factors such as diet in oncogenesis prevention 21. Relative to conventional cancer treatments, plant consumption containing highly effective phytochemicals can be less of an economic burden and inconvenience than surgery and chemotherapy. 22,23 Thus, phytochemicals can help address the critically important issue in healthcare economics, as rising medical costs make conventional treatments unaffordable for many segments of the population. Additionally, this serves as a non-invasive method of preventing cancer progression and reducing the size of tumors 20. For instance, the low incidence of bowel cancer in India is primarily attributed to the phytochemical curcumin, found in turmeric root, a spice commonly used in Indian cooking. A study in which high-risk patients for colon cancer received a dose of 480mg curcumin and 20 mg quercetin saw a significant decrease in the size of polyps in their colons 24.

In recent years, there have been many findings over the predictive targeted preventative medicinal effects of phytochemicals 25. Flavonoids have shown to combat the Warburg effect - elevated lactate secretion and glucose uptake in cancerous cells. 25 Other findings include those for certain phytochemical compounds such as curcumin in turmeric root 26, phytochemicals' efficacy for certain cancers such as pancreatic cancer 27, or the potential of phytochemical compounds in the alleviation of cancer treatment side-effects, such as isoflavones for chemotherapy-induced oral mucositis 28. Here, we will provide an extensive summary of the research status regarding phytochemicals' potential for chemoprevention and alleviation of chemotherapy-induced side effects.

In the following review, the side-effects of chemotherapy and chemotherapeutic drugs will be discussed. Furthermore, the bioavailability and efficacy of certain dietary phytochemicals (isoflavones, quercetin, curcumin, catechins, and hesperidin) in chemoprevention will be examined as well as the role of these phytochemicals in the alleviation of chemotherapy-induced side-effects. Conversely, gaps in the foundations of phytochemical research and further implications will be discussed.

## Side Effects of Chemotherapy and Chemotherapeutic Drugs

Although traditional chemotherapy is useful, it also entails many adverse effects. Many side effects of chemotherapy are dose-limiting, meaning their severity may cause a halt of cancer treatment. In both humans and animal models, chemotherapeutic drugs such as 5-FU, cisplatin, and doxorubicin can lead to neurotoxicity, gonadotoxicity, renal toxicity, hepatotoxicity, and other organ damages.

Chemotherapy-induced Nausea and Vomiting (CINV), caused by chemotherapeutic drugs, is among the most common side effects of chemotherapy treatment. Typically, the acute phase of CINV, which is moderated by 5-HT3 receptors in the intestine, occurs during the first twenty-four hours after chemotherapy administration 129. Chemotherapy causes free radicals to form, inducing serotonin release from cells in the intestinal mucosa 29. Serotonin then interacts with 5-HT3 receptors on intestinal wall nerves, exerting a vomiting reflex 29. Typically, CINV can be accurately predicted and contained during the first 24 hours after chemotherapy using 5-HT3 receptor antagonists, but symptoms may be delayed, affecting the quality of life 30. Delayed CINV usually starts 48 hours after chemotherapy administration and is mediated by a pathway with substance P 30. Substance P is a neuropeptide constituted of an eleven amino acid chain and is part of the tachykinin neuropeptide family, behaving like a neurotransmitter and neuromodulator 32. Chemotherapy causes substance P to be released from neurons and bind to NK-1 receptors in the medulla oblongata, inducing vomiting 33,34.

The mutagenic DNA damaging drugs used for chemotherapy cause skin and hair loss and have adverse effects involving bone marrow (e.g., anemia) and many other symptoms. Alkylating agents, a drug class used in cancer treatment, damages DNA by adding alkyl groups to DNA, inducing faulty nucleotide pairing, halting DNA transcription, and preventing DNA strand separation for reduplication due to cross-linking 35. Due to alkylating agents' carcinogenic nature, patients who are administered alkylating agents have a higher risk of a more treatment-resistant form of secondary cancer (e.g., acute non-lymphocytic leukemia) 35.

Antimetabolites, another class of chemotherapeutic drugs, inhibit metabolite use, a product of metabolism involved in growth and reproduction. Therefore, these drugs inhibit cell division and growth from occurring, which is useful for halting tumor growth 36. 5-fluorouracil (5-FU), a type of antimetabolite, is one of the most common chemotherapeutic drugs. However, recent studies have demonstrated that the administration of antimetabolites, such as 5-FU, can lead to side effects such as hair loss, photosensitivity, mood disorders, and mucositis 36-38. Cardiotoxicity, damage to heart muscles, caused by 5-fluorouracil (5-FU) is of great concern 39. Based on the drug dosage and drug administration method, heart muscle damage incidence ranged from 1-18% in patients treated with a fluoropyrimidine (a group of drugs, including 5-FU) 39. Although the explicit mechanism of 5-FU induced cardiotoxicity is not known, possible mechanisms include coronary vasospasm, myocardial injury, or impaired oxygen delivery 41. Coronary vasospasm, the constriction of coronary arteries causing reduced blood flow to the heart, causing ischemia (reduced blood supply to heart muscles), is the most studied mechanism of fluoropyrimidine induced cardiotoxicity 42. Coronary vasospasm likely attributed to the cardiotoxicity patients experienced after exposure to 5-fluorouracil and had high plasma levels of endothelin-1, a vasoconstrictor present in coronary artery disease 43. Typically, a higher rate of coronary vasospasm in patients results from a continuous infusion of the 5-FU drug rather than intermittent injections 43. 5-FU also led red blood cells to change confirmation from a biconcave to echinocyte shape (abnormal cell membrane with thorn-like projections), which caused a decline in red blood cells' ability to transport oxygen at the usual efficiency 42.

The next section examines the potential of five different phytochemicals for chemoprevention and alleviation of the side effects discussed above.

## Phytochemicals for Cancer Prevention and Alleviation of Chemotherapy Side Effects

### Isoflavones

Isoflavones, a type of polyphenol (a phytochemical extensively found in plant-based foods), are perhaps one of the most controversial groups of phytochemicals with potential for chemoprevention. Isoflavones are found in the Leguminosae family plants, such as soy, lentils, chickpeas, and beans 44. The isoflavones in soybean, such as daidzein, glycitein, and genistein, are known to have a large role in health 45. Genistein, an isoflavone in soy, is the most studied and shown to have the most positive impact on human health, as it has been shown to have anticancer effects against head and neck squamous cell carcinoma, breast, prostate, lung, cervical, ovarian, renal, liver, and bladder cancer 46-55. Even though isoflavones are in the inactive form in plants, known as glycosides, they convert to the active form, or aglycones, in the human intestine, as they are hydrolyzed by bacterial beta-glucosidase 56. The active form of isoflavones (aglycones) has a higher bioavailability than glycosides, meaning they would have a higher efficacy for chemoprevention in humans 57.

Several epidemiological studies have reported a 4-7 times higher breast cancer rate in the U.S. than China and Japan, which can be attributed to the high isoflavone content in the traditional Asian diet, which includes soy products as tempeh, tofu, soy sauce, and miso 59. Asian women with lower breast cancer prevalence had significantly higher levels of isoflavones in their plasma 59. In general, Soy isoflavones also have significant anti-angiogenic effects, shown through increased endostatin and decreased VEGF (vascular endothelial growth factor) levels, as well as a lower tumor microvascular density 60. Several studies have shown a much higher rate of prostate cancer in males in Western countries over Asian males, which suggests that the soy-rich foods in the traditional Asian diet potentially block prostate cancer 61. Genistein increased transcription of p21, a Cyclin-dependent kinase inhibitor, resulting in cell cycle arrest in prostate cancer cell lines PC-3 and LNCaP 62. In another case, both genistein and daidzein decreased cyclin B1 and increased p53 expression in LNCaP and PC-3 cell lines 63.

Although genistein sensitizes cancerous cells to conventional treatments such as radiotherapy and chemotherapy, genistein and daidzein are shown to protect non-cancerous cells from the toxic side-effects of conventional cancer treatment 64-66. One study comparing adverse effects of cancer treatment on treatment with and without soy isoflavone supplementation showed that it is an effective agent to combine with chemotherapy or radiation therapy to improve conventional treatment efficacy and reduce side effects caused by chemotherapeutic drugs and radiation 67. Children who took the soy isoflavone supplement endured a shorter neutropenia period, fewer infections requiring less antibiotic use, and less severe and shorter duration of oral mucositis 67. At higher concentrations, genistein's antiviral properties have helped reduce the frequency of viral infections in children, which is essential since cancer patients often experience severe viral infection symptoms because of a suppressed immune system caused by conventional cancer treatment 68.

### Quercetin

Quercetin (Qu), a type of flavonoid, is a phytochemical widely present in foods typically consumed daily. Quercetin is found in many fruits, vegetables, whole grains, and red wine, such as cranberries, apples, and onions 69. Quercetin is found in the Qu-glucose-conjugate form or Qu-glucoside form in plants 70. In humans, this form of quercetin is absorbed through enterocytes (absorptive simple columnar epithelial cells in the intestine), hydrolyzed into the Qu-aglycone form. Enterocytic transferases (Beta-Glucuronidase, Sulfotransferase, and catechol-O-methyltransferase) then convert the Qu-aglycone form into glucuronidated, sulfonated, and methylated forms, which are transferred to the liver, where a set of reactions convert these forms into Qu-3-glucuronide and Qu-3′-sulfate, which are the main compounds in human plasma originating from quercetin 71. The conjugated form of quercetin has a higher bioavailability than the unconjugated form in humans. The plasma concentration for the intake of the same amount of the conjugated and unconjugated forms was higher when quercetin was absorbed in the conjugated form 72.

**Figure 1:**
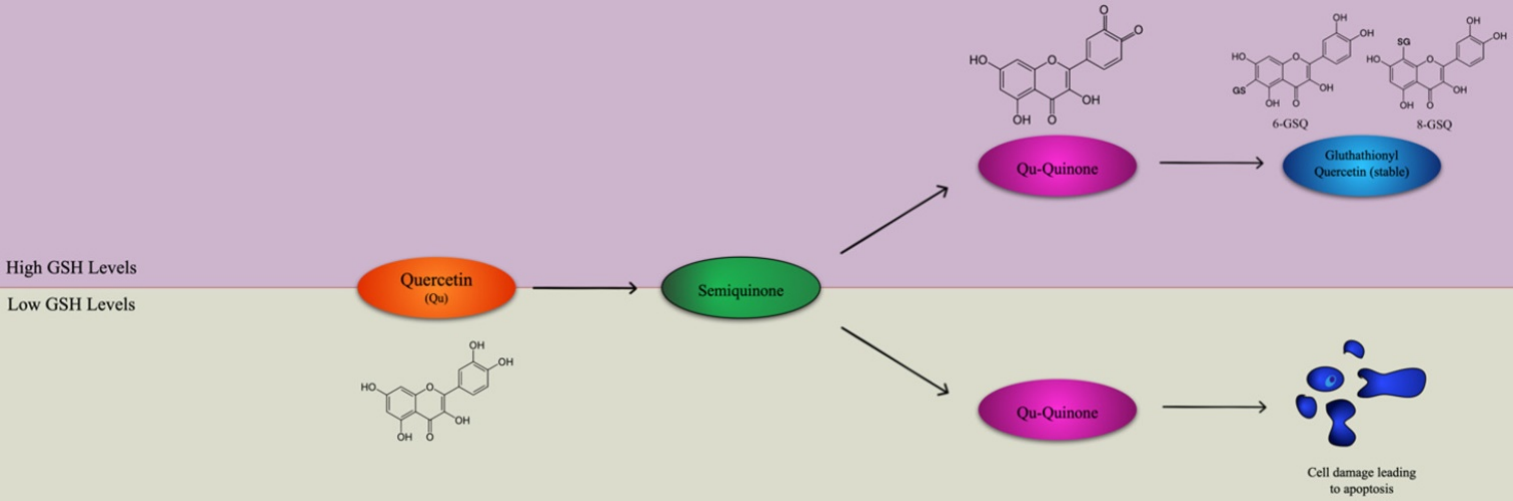
Quercetin applies either antioxidant or pro-oxidant effects depending on the level of intracellular reduced glutathione (GSH). Quercetin and H2O2 react to form a semiquinone radical which is oxidized to Quercetin-Quinone (Qu-Quinone). In greater levels of GSH, QQ reacts with GSH to form the stable glutathionyl quercetin (GSQ) forms of 8-GSQ and 6-GSQ. However, when there are lower levels of GSH, Qu-Quinone reacts with protein thiols, resulting in cell damage leading to apoptosis. Adapted from 75.

Quercetin has a high potential as a possible complementary treatment for many cancers such as ovarian cancer, prostate cancer, oral squamous cell carcinoma, because of its pro-apoptotic and anti-inflammatory properties 73. Although quercetin is an effective antioxidant due to its high number of hydroxyl groups and pi orbitals, it can also promote reactive oxygen species 74. Quercetin's primary oxidation product is semiquinone radical, which is unstable and goes through another reaction to produce the second reaction product, Qu-quinone (QQ), causing DNA and protein damage 74. However, whether quercetin exerts pro-oxidant or antioxidant effects depends on the levels of intracellular reduced glutathione (GSH) available 75. In an environment with elevated levels of GSH, Qu-quinone cannot induce cytotoxic effects on DNA and proteins because it reacts with GSH to produce glutathionyl quercetin (GSQ), a stable form of quercetin 75. DNA and protein damage occurs when there is a low level of GSQ because QQ reacts with protein thiols, leading to apoptosis, as shown in Figure 1 75. This property shows the potential of the medicinal use of quercetin for cancer treatment, along the lines of oxidation therapy 76. At the mRNA level, quercetin can increase p21 and p73 while decreasing the expression of cyclin B1, leading to the halt of the cell cycle at the G2-M checkpoint in esophageal squamous cell carcinoma cell lines 77. It was shown that even though quercetin treatment was significant, the dose of treatment that had led tumor cell lines to cell cycle arrest had little to no effect on normal, non-transformed cells 78. In a study where a K562 (myelogenous leukemia) cell line was treated with quercetin, the cells' growth was inhibited, and they were almost completely blocked from proliferation after 24 hours 79. After 48 hours, caspase 3, a protein with a central role in apoptosis, activity increased by 3.5 times, confirming that the quercetin treatment led to mechanisms for apoptosis 79. In the human breast cancer cell line MDA-MB-231, a low dose of the quercetin treatment inhibited proliferation of breast can 80. In oral squamous cell carcinoma, the epithelial to mesenchymal t promotes metastasis by increasing primary tumor cell motility and causing the loss of adhesion between the cancerous cells 81. In addition, matrix metalloproteinase (MMP), an extracellular protease, degrades the extracellular matrix which allows for the transport of cancerous cells into blood and lymph vessels 82. When oral squamous cell carcinoma lines HN22, SAS, and OSC20 were treated with quercetin, MMP-2 and MMP-9 activation decreased, exhibiting MMP proteases' accumulation due to quercetin 83. In the OSC20 and SAS cell lines, EMT activating transcription factors Slug and Twist were downregulated in cells treated with quercetin while in the HN22 cell line, Slug was downregulated in quercetin treated cells 83. Quercetin also exerts its anticancer effects by interfering with the Wnt/β-catenin cell signaling pathway. If not regulated, the WNT/β-catenin pathway accumulates β-catenin in the cell nucleus, which upregulates cyclin D1 and protooncogene c-myc 84. Quercetin was shown to inhibit Wnt/β-catenin cell signaling by downregulating cyclin D1 and survivin in SW480 colon cancer cells 85.

Although quercetin has been shown to be effective at chemoprevention, its activity is mainly exclusive towards transformed cell lines rather than indiscriminately inducing apoptosis and cell cycle arrest in tumors and standard cell lines 78. Quercetin can exert apoptotic potential on leukemic cell lines without causing apoptosis in peripheral blood mononuclear cells 86. Gastrointestinal mucositis, the ulceration and inflammation of the mucous lining of the gastrointestinal tract, occurs in up to 40% of patients going through chemotherapy 87. One study compared the effect of quercetin treatment on rats treated with methotrexate, a chemotherapeutic drug that is known to induce gastrointestinal mucositis 88. Rats treated with quercetin experienced decreased mucosal inflammation, while damage to the intestinal mucous lining was reversed 88. Even though quercetin induces apoptosis in many tumor cell lines, caspase-3 protein levels were lower in the rats treated with quercetin, demonstrating the anti-apoptotic effects quercetin exerted in intestinal cells during repair of the intestinal mucous lining 88. Chemotherapy-induced fatigue is one of the most common adverse effects of chemotherapy and is typically caused by the chemotherapeutic drug, 5-FU. A study conducted on two groups of mice treated with 5-FU showed that the group of mice treated with quercetin initially had low levels of activity following 5-FU treatment but returned to normal activity levels 8 days after 5-FU treatment. In contrast, mice without quercetin treatment returned to normal activity levels 14 days after 5-FU treatment 89. Typically, the 5-FU treatment causes an increase in Monocyte Chemoattractant Protein-1 (MCP-1) inflammatory cytokine in the plasma, which is associated with fatigue and is a marker of anemia 90. Quercetin treatment effectively crippled the response of MCP-1, as there was no change in MCP-1 levels after 5-FU treatment in mice created with quercetin compared to elevated levels of MCP-1 after 5-FU treatment in the group of mice without quercetin treatment 89. This can be attributed to the decrease in MCP-1 promoter gene expression and decrease in the activation of the Nuclear Factor Kappa B (NF-κB) pathway (key mediator in inflammatory response) by quercetin 91.

### Curcumin

Curcumin, a type of polyphenol, is a pigment found mainly in the rhizome of the plant Curcuma longa (Turmeric), but can also be found in Curcuma aromatica, Curcuma Mangga, Zingiber cassamunar, Etlingera elatior, Costus speciosus, Curcuma xanthorrhiza, Curcuma phaeocaulis, and Curcuma Zedoaria 92. Turmeric has been used in ancient Asian medicine for thousands of years to treat various inflammatory conditions and is a common spice used in Indian cooking 93. Although curcumin has poor bioavailability in humans due to its low solubility in water, many factors can increase the absorption of the phytochemical 94. Oral intake of the combination of curcumin and piperine (natural alkaloid of black pepper) significantly increased bioavailability and absorption of curcumin due to the inhibition of glucuronidation by piperine 95. The combination of curcumin and lecithin has been shown to improve gastrointestinal absorption of poorly water-soluble phytochemicals such as curcumin due to the amphipathic property of the phospholipids in lecithin 96. Nanoparticle drug delivery is one of the most promising methods for increasing drug bioavailability. Curcumin taken along with hydrophilic nanoparticles can increase oral absorption by 46 times due to increased water-solubility of the combination compared to pure curcumin 97, 98.

Many studies have illuminated on curcumin's chemopreventive effects due to its anti-inflammatory and antioxidant properties. One of the first studies on curcumin and cancer investigated curcumin's effect on cancerous lesions' on skin surface 99. In the clinical trial, 90% of patients experienced a decrease in the lesions' swelling, while 10% experienced a reduction in lesion size with a 1% curcumin ointment application 99. Curcumin decreased lipid peroxidation and restored the hepatic glutathione antioxidant defense, leading to the prevention of hepatic cancer in Wistar rats 100. In a clinical trial where 2 g of Meriva (contains curcumin phytosome complex) and 100 mg/m^2^ of gemcitabine (chemotherapeutic drug) were administered to 44 pancreatic cancer patients, the patients had an overall survival of 10.2 months compared to 5.7 months when treated with only gemcitabine 101. Curcumin typically accumulates in the colon's mucous lining compared to other areas of the body, hinting at curcumin's vital role colorectal cancer 102. In a clinical trial for colorectal cancer, oral intake of 3.6g of curcumin decreased prostaglandin E2 (a bioactive lipid that exerts many cancer and inflammatory-related effects) production in blood after 1 hour of administration 103. Another study done over 126 colorectal cancer patients showed a significant increase in tumor cell apoptosis after 1,080 mg of curcumin was given daily for 10-30 days, which can be attributed to the upregulation of p53 and increase in Bax/Bcl-2 ratio in the tumor tissues 104. Bcl-2 and Bax are the major proteins of the Bcl-2 family with roles in tumor progression 105. Bax is a pro-apoptotic protein that causes apoptosis by increasing mitochondrial membrane permeability, releasing cytochrome c into the cytosol 105. On the other side, Bcl-2 is an anti-apoptotic protein and inhibits Bax activity 105. Therefore, the ratio of Bax and Bcl-2 proteins present determines the fate of tumor progression.

**Figure 2:**
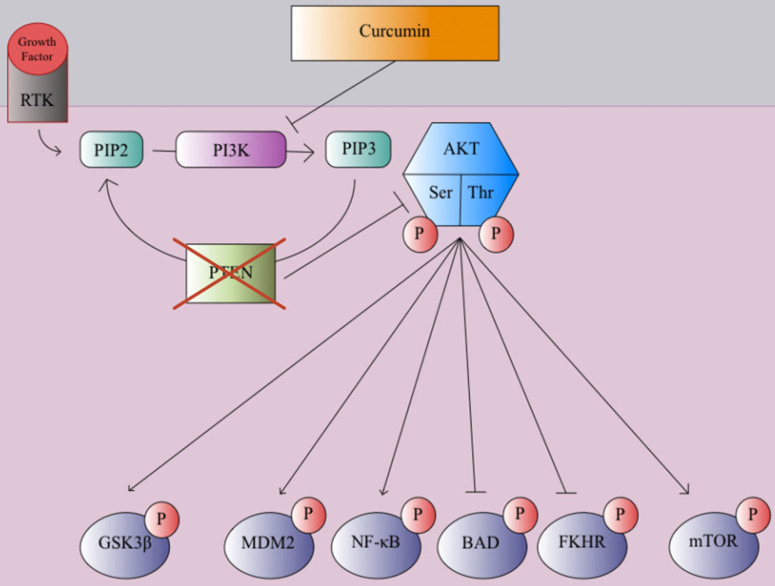
PTEN, a tumor suppressor and regulator of the PI3K/AKT pathway is commonly disrupted and unable to inhibit AKT phosphorylation during the early stages of cancer progression. Curcumin inhibits PI3K and blocks AKT phosphorylation which in turn blocks mTOR and MDM2 phosphorylation while preventing inactivation of GSK3β via phosphorylation and the suppression of pro-apoptotic and antiproliferative proteins, reducing cell growth, proliferation, and motility 121-123.

In many clinical trials and studies, curcumin has been shown to suppress cancer cell invasion through growth factor, cytokine, and adhesion molecule regulation 106. One mechanism of curcmin's antitumor property is the inhibition of the NF-κB pathway, which causes the prevention of MMP, inflammatory cytokines, and metastatic enzyme expression 107. Curcumin prevents the degradation of IκBα, which inhibits the NF-κB transcription factor 109. Genes associated with tumor formation are not expressed since the p65 subunit of NF-κB does not transport to the nucleus 109. This inhibitory process leads to apoptosis and decreased cell proliferation of various tumor cell lines 109. Curcumin plays a significant role in the downregulation of the WNT/β-catenin pathway to prevent cancer and tumor growth, as it has been shown to promote apoptosis and inhibit tumor cell migration in hepatocellular carcinoma cells by decreasing expression of glypican-3, which can inhibit c-myc, cyclin D1, VEGF, and β-catenin expression, which in turn inhibited WNT/β-catenin signaling 110-113. Curcumin can promote apoptosis and inhibit gastric carcinoma cell growth through suppression of Wnt3a, LRP6, phospho-LRP6 at Ser1490, β-catenin, phospho-β-catenin at Ser675, C-myc, and survivin proteins involved in the WNT/β-catenin pathway 114. Curcumin can also exert its anticancer effects through the modulation of the JAK/STAT signaling pathway. Once cytokines bind to cytokine receptors, the receptors are dimerized, phosphorylating JAK (Janus Kinase), which in turn, phosphorylates the cytosolic domain of the cytokine receptor 115. Eventually, the STAT protein is phosphorylated, forming a dimer, which transports to the nucleus and acts as a transcription factor to promote various cytokine-mediated immune responses 115. Misregulation of the JAK/STAT cell signaling pathway can lead to EMT and tumor growth 116. Curcumin can inhibit proliferation and promote apoptosis in retinoblastoma cells by reducing STAT1, STAT3, and JAK1 phosphorylation, suppressing the JAK/STAT pathway 117. Curcumin was also able to inhibit angiogenesis in laryngeal squamous cell carcinoma cells by inhibiting JAK2 and STAT3 phosphorylation, preventing VEGF and MMP-2 118. The PI3K/Akt pathway plays a large role in promoting cell growth, proliferation, and metabolism 119. Growth factors, such as EGF and VEGF, bind to RTKs, forming an active complex through phosphorylation 117. Then PIP2 converts to PIP3 through an intermediate PI3K, activating AKT after phosphorylation of its serine and threonine groups 119. The AKT protein promotes cell growth and proliferation through phosphorylation of mTOR and NF-κB, inactivation of Glycogen synthase kinase three beta (GSK3β) via phosphorylation, and blocking of apoptotic mechanisms through the phosphorylation of NF-κB, FKHR, BAD, and MDM2 119. Overexpression of the PI3K/Akt pathway can lead to cancer progression, as AKT kinase regulator PTEN is typically disrupted in many cancers 120. Phytochemicals such as curcumin exert antitumor effects on breast cancer cells through inhibition of PI3K phosphorylation, in turn, inhibiting mTOR and NF-κB phosphorylation and avoiding inactivation of GSK3β as shown in Figure 2 121-123.

In addition to the antitumor, anti-proliferative, and pro-apoptotic effects curcumin as on various cancer cell lines, curcumin has shown to have a preventative effect against chemotherapy-induced side effects 124. Chemotherapy-induced gastrointestinal damage can lead to the destruction of the intestine's mucous lining, causing higher vulnerability to viral infections, anorexia, nausea, vomiting, and fever; it can also be a dose-limiting factor reducing the efficacy of chemotherapy cancer treatment 124. In one study, 60 Wistar rats were divided into three groups, one treated with 5-FU only, another treated with 5-FU and curcumin, and the last group as a control 125. The 5-FU only treated rats experienced a destructed mucous lining, villi loss, infiltration of lymphocytes, and the death of the small intestine 125. When curcumin was administered along with 5-FU, the mucous lining was not as infiltrated with lymphocytes and was thicker with less destruction and villi loss 125. Curcumin can also make various tumor cell lines more sensitive to chemotherapeutic drugs such as cisplatin but protects non-transformed cells against cisplatin-induced neurotoxicity 125. In one study, curcumin reduced cisplatin-induced DNA damage in non-transformed PC12 cells 126. In a more recent study, curcumin reduced up to 50% of cisplatin's toxic inhibitory effect on neurite outgrowth in Wistar rats 127. Myelosuppression, the suppression, and decrease in bone marrow activity leading to a reduction in RBC production are common side effects of chemotherapy, especially the chemotherapeutic drug carboplatin 128. In a study conducted with a tumor-bearing mouse model, mice's survival rate was significantly higher when administered a combination of curcumin and carboplatin compared to only carboplatin over 20 days 128. The group of mice that were administered a combination of curcumin and carboplatin experienced improved bone marrow damage and an increase in white blood cell and platelet counts to levels before carboplatin treatment, illuminating on curcumin's ability to promote the repair of carboplatin-induced myelosuppression 128.

### Catechins

Catechins, a type of polyphenol, are a group of phytochemicals present in fluids such as green tea produced from the leaves of the Camellia sinensis plant 130. The primary catechin in green tea is epigallocatechin-3-gallate (EGCG), and consequently, the most thoroughly studied catechin 130. Other catechins present in green tea include epigallocatechin (EGC), epicatechin (EC), epicatechin-3-gallate (ECG), caffeic acid (CA), gallic acid (GA), gallocatechin (GC), catechin (C), and catechin gallate (CG) 130. Transferases catechol-O-methyltransferase, UDP-glucuronosyltransferases, and sulphotransferases conjugate a large fraction of tea catechins absorbed by the liver and small intestine, while microbes degrade the rest of the catechins into flavonoid rings after passing through the colon 131. These conjugated forms can circulate in plasma and perform biological functions after being distributed to the necessary tissues 131. Catechins are typically transported into cells through passive diffusion, as there are currently no known receptors on the intestinal epithelium that are known to assist in the transport of tea catechins into cells to perform biological functions 132. Multidrug-resistance associated protein efflux pumps such as P-glycoprotein pump out absorbed catechins into the intestinal space, limiting the bioavailability of tea catechins 133. In vitro studies have shown that around 1-100 μmol/L of ECGC is required to exert significant anticancer and anti-inflammatory effects, but due to the instability of EGCG in the GI tract and poor absorption, the plasma in humans had a low EGCG micromolar range 134. Nanoparticle drug delivery is the most promising method of increasing the bioavailability of catechins such as EGCG. Nano-carriers using carbohydrates, proteins, and lipids for tea catechins can improve solubility, increase permeability in the intestine to catechins, and decrease catechins' degradation in the small intestinal lumen 135,136. Modification of catechins' molecular structure, such as a peracetylated version of EGCG (AcEGCG), has been shown to increase bioavailability and stability in various internal conditions 137. Dextran-Catechin, a chemically conjugated catechin form, was more effective in counteracting neuroblastoma and drug-resistant tumor cells than unconjugated catechin 138. The synergistic combination of catechins and other chemotherapeutic drugs or phytochemicals (piperine, genistein, curcumin) has also been shown to increase the bioavailability and efficacy of dietary catechins in cancer treatment 139.

Tea catechins, most notably EGCG, have been proven to combat many cancers such as neuroblastoma, breast cancer, prostate cancer, and colorectal cancer 140,141. One study investigating the effects of EGCG on the cell growth of breast cancer cell lines MDA-MB-231 and MCF-7 found a dose of 100um of EGCG to decrease the cell viability index of MCF-7 and MDA-MB-231 by over 50% in 24 hours, suggesting the significant inhibition of MDA-MB-231 and MCF-7 cell line growth 142. A diet including Polyphenon E, a green tea polyphenol extract with a high concentration of catechins due to a water-based extraction method, decreased the amount of Benzo[a]pyrene-induced lung tumors in AJ inbred mice 143, 144. In a study investigating the effect of tea catechins on the SW-480 and HCT-116 colorectal cancer cell lines, phytochemicals in the galloylated catechin group exerted the most powerful anti-proliferative effects. 100um of EGCG, ECG, GCG, and CG inhibited HCT-116 cell growth by 98.4%, 20.3%, 79.2%, and 20.2%, respectively 145. Oral ingestion of Polyphenon E led to a significant decrease in the development of tumors in Tyrosine hydroxylase-MYCN transgenic mice, as half of the Polyphenon E treated mice were tumor-free after eight months, compared to the control group 146. EGCG treatment inhibited invasion and migration of Nasopharyngeal Carcinoma (NPC) cells, but only exerted a minor anti-proliferative effect on the NP460hTert immortalized NPC cell line 147.

Many cases have shown catechins' efficacy in inhibiting tumor cell proliferation, invasion, migration, and growth. One of the pathways through which tea catechins exert their antitumor effects is the Wnt/β-Catenin pathway. As previously discussed, the Wnt/β-Catenin pathway's dysregulation leads to tumor progression 84. Typically, without the Wnt signal, a complex involving GSK3β, adenomatous polyposis coli, and axin phosphorylates β-catenin leads to the degradation of β-catenin by a proteasome 148. This process prevents β-catenin from transporting to the nucleus, leading the upregulation of cyclin D1 and protooncogene c-myc 84. In the presence of the Wnt signal, membrane receptors activate and degrade GSK3β, causing β-catenin to accumulate in the nucleus and influence transcription 149. EGCG can inhibit GSK3β degradation and phosphorylation while promoting GSK3β expression, leading to a decrease in the amount of β-catenin in cancer cells 150. EGCG was able to reduce β-catenin transcription and mRNA levels in a keratin-forming tumor cell line while increasing the breakdown and ubiquitylation β-catenin 150. EGCG is also able to exert its anticancer effects by interfering with the JAK/STAT signaling pathway. As previously mentioned, the JAK/STAT pathway's dysregulation can lead to EMT and tumor cell proliferation. In oral cancer cells, EGCG reduced phosphorylation of serine and tyrosine of the STAT1 activator and inhibited JAK1 and JAK2 phosphorylation 151. EGCG inhibited c-Myb expression, which in turn reduced NF-κB and STAT3 signaling, eliminating the proliferation and invasion of ovarian cancer cells 152. Inhibition of c-Myb also reduced the resistance of ovarian cancer cells against the chemotherapeutic drug cisplatin 152. EZH2 is a protein that mediates the trimethylation of the 27th amino acid in histone 3 (H3K27), causing the suppression of the tissue inhibitor of MMP3 (TIMP3), leading to the degradation of the ECM, allowing cancer cells to transport through blood and lymph vessels 153. EGCG inhibited the trimethylation of H3K27 by EZH2, preventing the potential for prostate cancer cell invasion and migration 154. Signaling of transforming growth factor-β (TGFβ) is regulated by SMAD proteins, type 1 and 2 receptors, and TGFβ ligands 155. Misregulation of the TGFβ signaling pathway can lead to cancer progression through SMAD2/3 protein expression, promoting the expression of Snail and Slug transcription factors that induce EMT 156. EGCG was able to repress EMT and the invasive and migratory activity of the 8505C lung cancer cell line by inhibiting the phosphorylation of SMAD2/3 and downregulation of epithelial cadherin (E-cadherin). This adhesion molecule prevents the migratory activity of cancer cells 157. miR-485, a microRNA with tumor-suppressing properties, targets CD44, a glycoprotein involved in cell proliferation, migration, and angiogenesis. In A549 cisplatin-resistant cancer cells, CD44 was overexpressed while miR-485 was suppressed, but EGCG lowered CD44 levels by amplifying CD44 suppression by miR-485 158.

Tea catechins have also shown to have a protective effect against the effect of chemotherapy drugs on non-transformed cells in addition to their anticancer properties. Irinotecan (IT) is a chemotherapeutic drug with dose-limiting toxicity because of its severe effects on the small intestine 159. Irinotecan treatment caused high levels of inflammation and lipid peroxidation due to myeloperoxidase (MPO) activity, as well as a drop in GSH concentration of the ileum of mice and an increase in the oxidized version of GSH, glutathione disulfide (GSSG) 160,161. Low GSH levels lead to the activation of inflammatory pathways such as the NF-κB signaling pathway and an increase in MPO activity, leading to damage of non-transformed tissue 161. EGCG treatment before UV-radiation treatment in mice inhibited MPO activity and an increase in glutathione sulfide, lowering the GSH:GSSG ratio 161. EGCG was also able to inhibit leukocyte infiltration in IT-treated mice 162. However, more studies are needed on the correlation between green tea polyphenols and an increase in GSH concentration in the small intestine after irinotecan treatment. Doxorubicin (DOX), a chemotherapeutic drug in the anthracycline family, is effective against many types of cancers such as leukemia and breast cancer 163. However, doxorubicin can exert its gonadotoxic effects on human ovarian tissue, causing the loss of reproductive function in female patients 164. When EGCG was given in combination with doxorubicin, doxorubicin-induced inflammation in ovarian tissue was significantly reduced 165. This can be attributed to the inhibitory effect on doxorubicin-induced cytokine, interleukin, and MMP expression by EGCG 165.

### Hesperidin

Hesperidin, one of the most consumed flavonoids, is a phytochemical widely present in citrus fruits such as orange, tangerine, lemon, lime, and grapefruit, as well as herbs such as peppermint 166. Hesperidin is converted to its aglycone form, hesperidin, upon consumption 167. Hesperidin, a rutinoside, is hydrolyzed by the gut flora and then is absorbed in the colon 168. The peak plasma levels of hesperidin in relation to the amount of hesperidin ingested are similar to other flavonoids such as quercetin, but the absorption of hesperidin is lower than that of genistein, daidzein, and other isoflavones 167. Due to the gut microflora enzymes cleaving the C-ring from hesperidin, studies investigating urinary recovery determined hesperidin's low bioavailability 168. To reap effects from hesperidin, citrus foods must be consumed regularly because of the high urinary elimination rate of hesperidin. After 24 hours of hesperidin intake, 98% of the metabolites were eliminated in the urine, and hesperidin was not present in the plasma 167. Further studies are required to investigate methods to increase the absorption and bioavailability of hesperidin and other rutinosides.

Many studies indicate that hesperidin possesses high potential as a chemopreventive treatment for skin and colon cancer treatment due to its pro-apoptotic and anti-proliferative properties 169. Hesperidin was able to induce cell death of the HepG2 liver cancer cell line in a dose-response manner 170. In one study which investigated the effect of orange juice, which contains a large amount of hesperidin, on colon cancer, oral intake of orange juice reduced the extent of tumor growth in rats by 22% 171. A greater-strength orange juice was able to inhibit mammary cancer progression in rats 172. One study investigated the effect of hesperidin (aglycone form of hesperidin) on rats with 1,2-dimethylhydrazine (DMH) induced colon cancer 173. Rats in the control group (DMH administration without hesperidin) experienced an increased presence of aberrant crypt foci (ACF; a precursor to colorectal cancer) and tumor growth, as well as a decrease in glutathione peroxidase and glutathione S-transferase, a phase II detoxification enzyme 175-177. DMH-induced colon cancer rats experienced the most significant decrease in tumor and ACF formation when treated with a hesperidin dose of 20 mg/kg 173. Hesperidin treatment led to the suppression of lung carcinoma, neoplastic lesions, oxidative stress, and lipid peroxidation caused by benzo(a)pyrene in Swiss albino mice 178. Hesperetin-7-O-acetate, the acetylated version of hesperidin, significantly decreased cell viability and increased caspase-3 and caspase-9 activity of the A-494 renal carcinoma cell line 179. When the PC3 prostate cancer cell line was exposed to hesperidin, the cell viability decreased by 80% 48 hours after treatment, indicating the anti-proliferative effect of hesperidin 180.

One mechanism of Hesperidin's antitumor property is the inhibition of the specificity protein 1 (Sp1) transcription factor. The Sp1 transcription factor can regulate cyclin D1, survivin, Mcl-1, p27, and p21 gene expression, which are involved in cell growth, apoptosis, metabolism, and angiogenesis 181. Elevated levels of Sp1 have been associated with many cancers, indicating that Sp1 can potentially serve as a molecular target for cancer treatment and chemoprevention 182. Hesperidin's inhibitory effect on Sp1 promoted apoptosis in MSTO-211H, a human mesothelioma cell line 183. It upregulated Bax expression while downregulating Bcl-2 expression, disrupting tumor progression in the SNU-C4 human colon cancer line 184. It has been able to exert its anticancer effects through inhibition of the NF-κB signaling pathway and activator protein 1 (AP-1), which in turn inhibits MMP-9 expression in the HepG2 liver cancer cell line 185. Hesperidin exerted pro-apoptotic effects by upregulating p53 and caspase-3 levels while inducing DNA fragmentation in the MCF-7 breast cancer cell line 186. It also exerts its pro-apoptotic effects by inhibiting the TGFβ signaling pathway by blocking the phosphorylation of SMAD3, thus leading to a reduction in cancer cell proliferation and metastasis 187. Hesperidin inhibited cell migration and tube formation in human umbilical vein endothelial cells due to the inhibition of the PI3K/Akt signaling pathway by hesperidin, blocking mTOR phosphorylation 188.

Although many studies have shown hesperidin to effectively block cell growth, proliferation, and metastasis promoting pathways in various cancer cell lines, hesperidin has a protective effect against chemotherapy-induced side effects. As discussed previously, cisplatin, a common chemotherapeutic drug, causes many adverse effects in patients such as gastrointestinal, neutrality, and CINV. In one study investigating the effect of hesperidin on cisplatin-induced eye toxicity in rats, hesperidin treatment of 50 mg per kg of body weight prevented retina and cornea damage, a drop in GSH levels, an increase in thiobarbituric acid reactive substances, and a decrease in antioxidant activity 189. Cisplatin-induced liver toxicity was alleviated by hesperidin without a decrease in anticancer activity in rats 190. Diarrhea is one of the most common adverse effects of chemotherapy and occurs in up to 80% of patients, while severe diarrhea occurs in more than 30% of patients, resulting in malnutrition and low quality of life 191. Hesperidin can reduce intestinal inflammation by blocking cytokine release, inhibiting NF-κB expression, and reducing inflammatory cell infiltration in an animal model with irinotecan-induced diarrhea 192.

## Conclusions and Outlook

Due to the prevalence of cancer incidence throughout the world, effective means of attenuating various cancers are of great interest. Although conventional chemotherapy is effective against many cancer cell lines, its non-specificity can result in various dose-limiting side effects. Chemoprevention by dietary phytochemicals is a relatively non-invasive targeted approach with predictive and preventative attributes 193, 194. In addition, phytochemical application has shown to be relatively cost-effective and more economically attractive in relation to conventional treatments. 25 In many clinical trials, phytochemical application inhibited various cell signaling pathways associated with cancer cell progression and protected normal cells against chemotherapy-induced damage. Nano Drug Delivery, an emerging technology, is a promising method for increasing the absorption and bioavailability of many dietary phytochemicals. Specific combinations for select phytochemicals such as piperine and curcumin. Despite the many advantages of phytochemicals reviewed in this paper, much of the evidence is based on early-stage research on mice. Additional clinical trials on humans have the potential to fill the gaps in phytochemical research and significantly increase our understanding of their potency, toxicities, efficacy, and drug-drug interactions.

This review has attempted to provide a summary of current research on phytochemical application as a complementary approach to chemoprevention. Understanding phytochemicals' effect on cancerous cells and surrounding nontransformed cells will allow for a more cost-effective, non-invasive, and targeted approach to cancer treatment in the future.

## Figures and Tables

**Table 1:** Summary of various phytochemicals' efficacy in chemoprevention and alleviation of chemotherapy-induced side effects

Phytochemical	Type	Source	Cancers	Attenuating adverse effects	Methods to increase bioavailability
Isoflavones	Polyphenol	Plants of leguminosae e.g. soy, chickpeas, lentils, beans	Head and neck cell carcinoma, breast, prostate, lung, cervical, ovarian, remal, liver, bladder	Neutropenia, Increased Vulnerability to viral infections	Nano Drug delivery technology, using active form of isoflavones (aglycones)
Quercetin	Flavonoid	Apples, onions, cranberries, red wine, whole grains	Ovarian, prostate, oral squamous cell carcinoma, esophageal squamous cell carcinoma	Gastrointestinal toxicity, Neurotoxicity	Using conjugated form of quercetin, nano drug delivery technology
Curcumin	Polyphenol	Rhizome of *Curcuma longa* (Turmeric)	Hepatic carcinoma, colorectal cancer	Gastrointestinal toxicity, Neurotoxicity, Myelosuppression	Nano drug delivery, piperine, lecithin
Catechins	Polyphenol	Green tea	Neuroblastoma, breast cancer, prostate cancer, colorectal cancer	Gastrointestinal toxicity, Gonadotoxicity	Nano drug delivery, molecular modification, piperin
Hesperidin	Flavonoid	Citrus fruits e.g. orange, tangerine, lemon, lime, grapefruit, herbs e.g. peppermint	Skin, colon, lung, renal carcinoma	Gastrointestinal toxicity, eye toxicity, hepatotoxicity	Further studies required
